# In vivo and in vitro characterization of a new Oya virus isolate from *Culicoides* spp. and its seroprevalence in domestic animals in Yunnan, China

**DOI:** 10.1371/journal.pntd.0011374

**Published:** 2023-06-15

**Authors:** Jinxin Meng, Fei Wang, Yuwen He, Nan Li, Zhenxing Yang, Jun Yao, Shunlong Wang, Guodian Xiong, Zhiming Yuan, Han Xia, Jinglin Wang

**Affiliations:** 1 Yunnan Tropical and Subtropical Animal Virus Disease Laboratory, Yunnan Animal Science and Veterinary Institute, Kunming, China; 2 Key Laboratory of Special Pathogens and Biosafety, Wuhan Institute of Virology, Chinese Academy of Sciences, Wuhan, China; 3 University of Chinese Academy of Sciences, Beijing, China; 4 Hubei Jiangxia Laboratory, Wuhan, China; The University of Hong Kong, CHINA

## Abstract

Biting midges are one of the most common hematophagous insects. They are capable of transmitting a wide range of arboviruses and have a significant impact on public health and veterinary medicine. Herein, from midge samples collected in 2013 in Yunnan, China, one sample induced a cell cytopathic effect (CPE) in BHK-21, MA104, and PK15 cell lines. Next-generation sequencing data, RACE and PCR determined the genome sequence of the sample and designated as an Oya virus (OYAV) isolate SZC50. Phylogenetic analysis of the sample revealed that it was cluster into viruses from species *Orthobunyavirus catqueense*. The open reading frames of S, M, and L segment of OYAV SZC50 were closest to those of OYAV SC0806. Moreover, 831 serum samples (736 pigs, 45 cattle, and 50 sheep) were gathered from 13 cities in Yunnan Province to detect neutralizing antibody of OYAV SZC50. A significant proportion of OYAV SZC50 antibody (more than 30%) was found in Yunnan pig populations, with the positive rate of OYAV SZC50 antibody in pigs from Malipo reaching 95%. To determine the pathogenicity of OYAV SZC50, we chose three animal models: specific pathogen-free Kunming mice, C57BL/6 mice lacking the interferon α/β receptor, and chicken embryos. At 5, 6, and 7 days post-infection, all adult and suckling C57BL/6 mice, and specific pathogen-free suckling Kunming mice were dead. Our finding was expanding the knowledge about the infection and pathogenic risk of the neglected virus in the *Orthobunyavirus*.

## Author summary

*Culicoides* are an important component of arbovirus ecology and should be studied and watched over in order to the potential spread of emerging and re-emerging viruses. A novel OYAV isolate (SZC50), isolated from midge samples was sequenced in this study, and the virus’s putative host tropism was also tested in vitro, and cell lines derived from pig showed a highly susceptible to OYAV SZC50. Notably, high prevalence of OYAV SZC50 neutralization antibody was found in Yunnan pig serum samples. Moreover, the pathogenicity of OYAV SZC50 in animal models was first shown using mice and chicken embryos. Adult C57BL/6 and suckling mice were shown to be extremely sensitive to OYAV SZC50 infection, in contrast to adult Kunming mice, who can survive following infection. The maximum fatality rate of chicken embryos was 37.5%. In summary, regarding the potential infection risk of OYAV to animal or human, systematic investigation of OYAV, its vector and reservoir in nature should be conducted.

## 1. Introduction

The extensive global spread of arbovirus has been a key cause for concern during the past few decades [1]. As the vector of arbovirus disease, biting midges (Diptera: Ceratopogonidae) are among the most common hematophagous insects. They have a significant influence on public health and veterinary medicine. In many areas, the midges act as vectors to transmit a variety of pathogens to humans and both domestic and wild animals, notably arboviruses to domesticated animals [2]. To date, more than 50 arboviruses have been identified from *Culicoides* spp. Several cause illnesses of such worldwide importance that they are included in the Listed diseases designation by the World Organization for Animal Health (WOAH) [3]. Among them, orthobunyaviruses of the Simbu serogroup are presumably the most important group as they are evidently transmitted by *Culicoides* midges. Its members are particularly significant in veterinary medicine; viruses in the Simbu serogroup, including *Akabane orthobunyavirus* (AKAV) and *Schmallenberg orthobunyavirus* (SBV), cause devastating congenital diseases in pregnant animals [4].

Yunnan Province in southwestern China shares a 4,061-kilometer border with Myanmar, Laos, and Vietnam [5]. The climate in this region is tropical to subtropical in the south with high temperatures, heavy rain, and high humidity. Yunnan Province has a diverse range of *Culicoides* vectors (at least 36 species) [6], which facilitates the spread of arboviruses [7]. Previous studies reported the detection or isolation of several arboviruses, including bluetongue virus (BTV) [8], Tibet orbivirus (TIBOV) [9], and Banna virus (BAV) [10] from *Culicoides* in Yunnan Province. The application of sequencing technologies has created a map of viromes of *Culicoides* and mosquitos in this area. The many novel viruses that have been found include 21 segmented viruses of *Flaviviridae*, 180 viruses of *Monjiviricetes*, and 130 viruses of *Bunyavirales* [11]. Therefore, *Culicoides* is a crucial component of viral ecology and it should be researched and monitored to identify novel viruses.

*Orthobunyavirus catqueense*, which comprises Cat Que virus (CQV) and Oya virus (OYAV), is a species of *Orthobunyavirus* genus in the family *Peribunyaviridae* [12]. Several viral isolates of this species have been identified in China, Vietnam, India, and Malaysia from invertebrate (*Culex* mosquitos and *Chironomus* midges) and vertebrate (pigs, birds, and humans) hosts [13–17]. The CQV is widely distributed in mosquitos and pigs; the extensive distribution of CQV strain SC0806 has been demonstrated through the positive results of SC0806 antibodies in pig populations obtained from several local regions of China [16]. OYAV was initially isolated from a pig suspected of Nipah virus infection during the National Swine Surveillance Programs for the Nipah Virus in Malaysia in 2000 [14]. It has been subsequently found in some mosquitos and midges [17]. The prevalence of OYAV in vertebrate hosts is still unclear with only one human sample and one pig sample are reported for this virus [13,14].

Here, we report a new isolate of OYAV (SZC50), isolated from midge samples collected in 2013 in Yunnan, China. The OYAV SZC50 genome was sequenced and the evolutionary relationships between OYAV SZC50 and other members of the *Orthobunyavirus* genus were analyzed. We also assessed the seroprevalence of OYAV SZC50 antibodies in pig, cattle, and sheep populations. Specific pathogen-free (SPF) Kunming mice, C57BL/6 mice, and chicken embryos were used as the models to assess the pathogenicity of OYAV SZC50.

## 2. Materials and methods

### 2.1. Ethics statement

Animal studies were approved by the Ethical Review Committee of Yunnan Animal Science and Veterinary Institute (Approve No. 2019FA015). All animal procedures were performed in strict compliance with the guidelines of Guide for the Care and Use of Laboratory Animals [18].

### 2.2. Cell lines and animals

*Aedes albopictus* C6/36 cells were cultured in Roswell Park Memorial Institute (RPMI) 1640 medium supplemented with 1% penicillin/streptomycin and 10% fetal bovine serum at 28°C in 5% CO_2_. BHK-21 (golden hamster kidney cells), MA104 (rhesus monkey kidney cells), PK15 (pig kidney cells), MDBK (bovine kidney cells), HeLa (human cervical cancer cells) and GOATTE (goat testis cells) were cultured in MEM supplemented with 1% penicillin/streptomycin (P/S) and 10% fetal bovine serum (Gibco; Thermo Fisher Scientific) at 37°C in 5% CO_2_.

SPF Kunming mice, C57BL/6 mice lacking the interferon α/β receptor [19], and chicken embryos [20] were employed as viral infection animal models. The mice were obtained from Kunming Medical University and kept in an ABSL-2 facility with regulated temperature (24°C), humidity (60%), and a 12:12 h light/dark cycle. The chicken embryos were obtained from Jinan Xinshengda Biological Engineering Company Limited and kept in an ABSL-2 facility with regulated temperature (37.5°C), humidity (70%), and a 12:12 h light/dark cycle.

### 2.3. Collection of midge specimens and serum specimens from animals

In July 2013, midges were collected at Longburui cattle farm in Wulong Township, Zong County, Yunnan, using a mosquito lure lamp (Gongfu Xiaoshuai, Wuhan, China) operating at 12 V and 300 mA. The sampling time was from 18:00 to 8:00 of the following day [21]. The collected midges were frozen at -20°C for 30 min and the other insects were removed. Approximately 50 midge specimens were packed into one tube. Each tube was numbered and stored in liquid nitrogen tanks for transportation to laboratory.

Blood samples of livestock, including cattle, sheep, and pigs, were collected from 13 sampling sites (Shizon, Shuangjiang, Yimen, Wuding, Maguan, Tengchong, Longling, Fuming, Dehong, Fengqing, Honghe, Fuyuan and Malipo) in various prefectures in Yunnan. These sampling sites covered most of the livestock breeding sites in Yunnan. Serum samples were separated in the laboratory and then frozen at -20°C until required.

### 2.4. Virus isolation and complete genome sequencing

The subpackaged midge specimens recovered from storage were added to a grinding solution (0.5 mL for approximately 50 midge specimens), and ground in a TissueLyser II high-throughput tissue grinder (QIAGEN, Valencia, CA, USA) precooled at -20°C for 10 min at the speed of 30/s. After centrifuging at 12,000 × g for 10 min, the recovered supernatant was used to inoculate a single layer of BHK-21 cells. The infected cells were observed daily; after 7 days, the supernatant was collected and used to inoculate fresh BHK-21 cells. Blind passage was sustained for three generations until a cytopathic effect (CPE) was observed in the cells. The supernatant of samples displaying CPE was used for expanded cultivation and was stored at -80°C for identification.

RNA extracted from the supernatant of inoculated cell cultures was sequenced using the HiSeq 2500 platform (Illumina, San Diego, CA, USA) by Nextomics Bioscience Co., Ltd. (Wuhan, China). The analysis and assembly of the sequencing data were performed with a self-designed and previously reported bioinformatics pipeline [22]. Virus-specific 5´ and 3´ rapid amplification of cDNA ends (RACE) primers (S1 Table) were used to confirm the complete genome sequence. The acquired complete genome of OYAV SZC50 was submitted to Genbank.

### 2.5. Phylogenetic analysis

The viral open reading frames (ORFs) were predicted using open-source NCBI ORF finder (https://www.ncbi.nlm.nih.gov/orffinder/). Multiple alignments of nucleic acid and amino acid sequences were performed using the MAFFT and MUSCLE webservers, which were edited by open-source Jalview software version 2.11.2.2 [23,24]. The phylogenetic tree was constructed using IQ-TREE multicore version 2.2.0 and the maximum likelihood method of 5000-fold ultrafast bootstrap [25,26]. The tree was subsequently modified by iTOL (https://itol.embl.de/upload.cgi). Virus abbreviations and accession numbers of selected viruses from Simbu and Bunyamwera serogroups orthobunyaviruses are listed in S2 Table.

### 2.6. Virus proliferation curves of SZC50 in various cell lines

The virus solution (multiplicity of infection [MOI] = 0.01) was inoculated into BHK-21, MA104, PK15, MDBK, Hela, C6/36 and GOATTE cell lines that were individually cultured in a 75 cm^2^ cell flask. Each cell line was cultured three times. For each cell line, cells that were not exposed to virus were included as a mock control. Cell culture supernatant was collected every 24 h from 0 to 120 h after viral infection and temporarily stored at -80°C. The 50% tissue culture infectious doses (TCID_50_) of the collected samples at each time point were determined by the plaque counting method [27]. The virus proliferation curve of virus in each cell line was plotted using GraphPad Prism 9.1.0 (GraphPad, San Diego, CA, USA).

### 2.7. Transmission electron microscopy (TEM)

The supernatants of infected BHK-21 cells (approximately 200 mL) were centrifuged at 125 × *g* for 10 min at 4°C to concentrate the virions. Each supernatant was filtered through a 0.22 μm membrane filter and transferred to an SW32 ultracentrifuge tube containing a continuous sucrose density gradient solution (30%−70%). Ultracentrifugation was performed at 31,000 × *g* at 4°C for 3 h. The pellet was immediately resuspended in 100 μL phosphate-buffered saline (PBS) and filtered using a 0.5 mL ultracentrifuge filter (Amicon® Ultra; Millpore, Billerica, MA, USA). The retained virions were suspended in PBS before being distributed in drops on Formvar-coated copper grids and stained with 2% uranyl acetate. Excess stain was removed in the standard manner and TEM was performed using a Tecnai G2 20 TWIN microscope operating at an accelerating voltage of 200 kV (FEI, Hillsboro, OR, USA).

### 2.8. Plaque assay

With reference to Alan Baer *et al*. [28], the plaque assay was briefly described below. Ten-fold serial dilutions (10^−1^–10^−7^) of OYAV SZC50 virus suspension were prepared using MEM. The diluted virus solution was inoculated onto BHK-21 cell monolayers in wells of 24-well plates for 1 h. After that, the cells were covered with an overlay medium including 1.5% methylcellulose and incubated at 37°C for 3−4 days to facilitate plaque formation. The infected cells were fixed overnight with 750 μL of 3.7% formaldehyde and stained for 5 min at room temperature with 2% Crystal Violet in 30% methanol. The plaques were manually counted and measured.

### 2.9. Viral neutralization antibody and nucleic acid detection

Serum samples collected from cattle, sheep, and pigs were subjected to antibody detection by the neutralization test [29]. Each serum was diluted 1:20, 1:40, 1:80, 1:160, 1:320, 1:640, and 1:1,280. Each serum dilution was mixed with an equal volume of virus supernatant (100 TCID_50_/50 μL). One hundred microliters of each preparation were inoculated into duplicate wells of 24-well plates. Viral supernatants of 100TCID_50_, 10TCID_50_, 1TCID_50_, and a blank control were set as the controls. The plates were shaken for 3–5 min and neutralized for 1 h at 37°C in a 5% CO_2_ incubator. After that, 100 μL aliquots of BHK-21 cell suspension (1 × 10^6^ cells/mL) was added to each well of 24-well plates. These plates were cultivated at 37°C in a 5% CO_2_ incubator. After 7 days of continuous observation, both the cells in blank control wells and cells treated with viral supernatants of 1 TCID_50_ grew normally, CPE was evident in all the cells treated with viral supernatants of 100 TCID_50_, and CPE was evident in some cells treated with viral supernatants of 10 TCID_50_. Based on these results, the observation time of viral neutralization antibody assay was set to 5−7 days. If the CPE was not observed in more than one well of cells, the titer of the particular serum dilution was the neutralizing antibody titer of this serum. The serum was determined as virus positive when the neutralizing antibody titer was ≥ 1:20. The positive rates of serum neutralizing antibody were plotted using GraphPad Prism 9.1.0 software. Differences were analyzed by Fisher’s exact test.

Viral RNA detection was performed on the collected serum samples of cattle, sheep, and pigs by qRT-PCR. Total RNA of serum samples (200 μL) was extracted by using viral nucleic acid extraction reagent (Tianlong Science and Technology, Xi’An, China). qRT-PCR of each sample was performed using One-Step TB Green PrimeScript RT-PCR Kit II (Perfect Real Time; TaKaRa Bio, Shiga, Japan) using the ABI 7500 Fast Real-Time PCR System (Applied Biosystems, Foster City, CA, USA). The reaction conditions were 42°C for 5 min, followed by 45 cycles of amplification (95°C for 10 s, 95°C for 5 s, and 60°C for 34 s). The primers for qRT-PCR were SZC50_228F: 5´-CCATTTTCCACAATTCCAAT-3´ and SZC50_339R: 5´-GACTGGGCTCTGGGCATAG-3´.

### 2.10. Experimental infection of OYAV SZC50 in animal models

#### 2.10.1. Viral infection in suckling and adult Kunming and C57BL/6 mice

Each suckling Kunming and C57BL/6 mouse was inoculated with 30 μL OYAV SZC50 solution (100 plaque forming unit [PFU]/100 μL) in the brain (the lesser viruses input for these mice whose immune systems are relatively fragile). The control groups were inoculated with the same amount of MEM. Each group consisted of 12 virus treated animals and two control animals. The symptoms of suckling mice and chicken embryos were monitored daily, and the time of death was recorded.

Each adult Kunming mouse (3-month-old) was inoculated intraperitoneally with 500 μL OYAV SZC50 (100 PFU/100 μL). Each control mouse was inoculated with 500 μL MEM. Each group consisted of six adult mice. Symptoms were monitored daily and the time of death was recorded.

Each adult C57BL/6 mouse was inoculated intraperitoneally with OYAV SZC50 (300 μL, 100 PFU/100 μL). Each control mouse was inoculated with 300 μL MEM. Each group consisted of three adult mice. Symptoms were monitored daily and the time of death was recorded. Pathological sections were generated to examine the pathological changes of tissues and organs of adult C57BL/6 mouse died on day five.

#### 2.10.2. Viral infection in chicken embryos

Chicken embryos (11-day-old) were inoculated with 100 μL of various viral titers of SZC50 solution in the allantoic cavity. Viral titers were set as 10^4^, 10^3^, and 100 PFU/100 μL. The control group was inoculated with the same amount of MEM. Each group consisted of eight chick embryos. Symptoms were monitored daily and the time of death was recorded. After 8 days, the chicken embryos were fixed with 3.7% formaldehyde at 4°C overnight. Pathological sections were generated to examine the pathological changes of tissues and organs of the chicken embryo died on day four.

Curves of survival rate of viral infected suckling and adult Kunming and C57BL/6 mice, and chicken embryos were plotted using GraphPad Prism 9.1.0 software.

## 3. Results

### 3.1. Infection of various cell lines by midge sample SZC50, with highest amplification efficiency in PK15 cells

**Fig 1:**
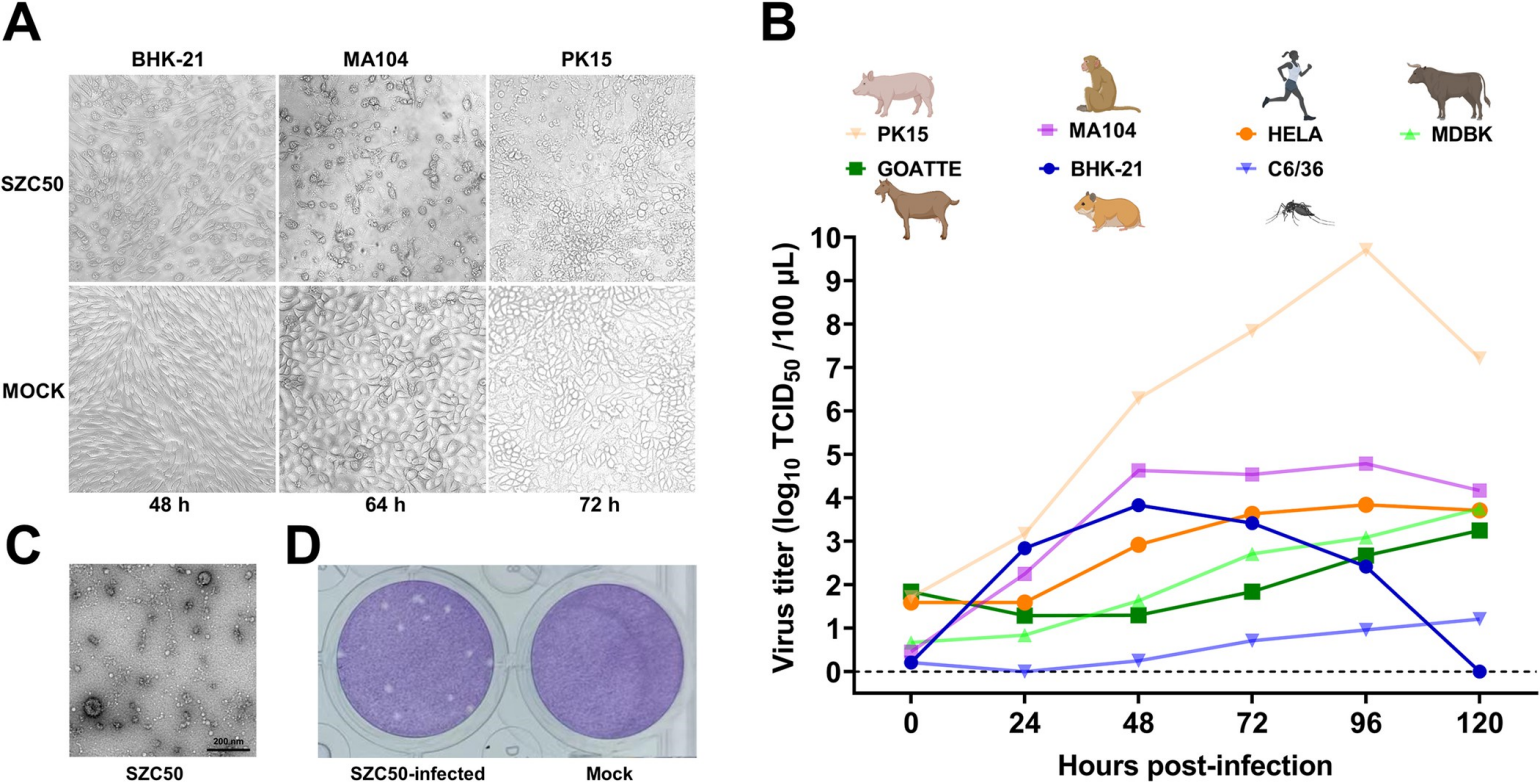
Appearance of SZC50 infected cells, SZC50 growth curves (MOI = 0.01) in cells derived from mammals and mosquitos, and cell morphology and viral plaque in BHK-21 cells are presented. (A) BHK-21 cells at 48 hpi, MA104 cells at 64 hpi, and PK15 cells at 72 hpi following SZC50 inoculation. (B) SZC50 growth curves in BHK-21, MA104, PK15, MDBK, Hela, C6/36, and GOATTE cell lines. (C) Negative-stained ultracentrifuged virions of SZC50. (D) Plaques generated by SZC50 in BHK-21 cell monolayers stained with crystal violet at 72 hpi. Cartoons of humans and animals from the BioRender site that were used in academic articles under a sublicense. Created with Biorender.com.

A total of 2,437 midges were captured at Longburui cattle farms in Wulong Township, Shizong County, Yunnan, in July 2013. One sample (SZC50) from 49 pools induced CPE in BHK-21 cells. Cells became spherical and detached after 48 hours (Fig 1A). To survey the different cellular infection profiles, the fifth generation of the supernatant from BHK-21 cells infected with SZC50 were inoculated into MA104, PK15, MDBK, Hela, C6/36 and GOATTE cell lines. CPE was observed in MA104 cells at 64 h post-infection (hpi) and in PK15 cells at 72 hpi (Fig 1B). CPE was not evident in the other cell lines.

Amplification of SZC50 in BHK-21, MA104, PK15, MDBK, Hela, C6/36 and GOATTE cells was investigated. Growth kinetics results indicated the amplification efficiency of SZC50 was highest in PK15 cells, with the highest virus titer of 10^9.71^ TCID_50_/100 μL at 96 hpi at an MOI = 0.01. Virus replication was lowest in mosquito C6/36 cells (the highest virus titer was 10^1.21^ TCID_50_/100 μL at 120 hpi at an MOI = 0.01). The highest virus titers in the other five cell lines ranged from 10^3.25^ to 10^4.79^ TCID_50_/100 μL at an MOI = 0.01. Notably, the viral titer in BHK-21 cells rapidly declined, with 10^3.83^ TCID_50_/100 μL at 48 hpi and virus inactivation after 120 hpi (Fig 1B).

To examine SZC50 particle morphology, BHK-21 cell culture supernatants infected with SZC50 were ultracentrifuged and examined by TEM. Spherical virus-like particles 80–90 nm in diameter were observed (Fig 1C). On the third day following inoculation, discrete, ragged plaques approximately 0.6 mm in diameter were evident in BHK-21 cell monolayers (Fig 1D).

### 3.2. Complete genome sequencing and analysis of OYAV SZC50

**Fig 2:**
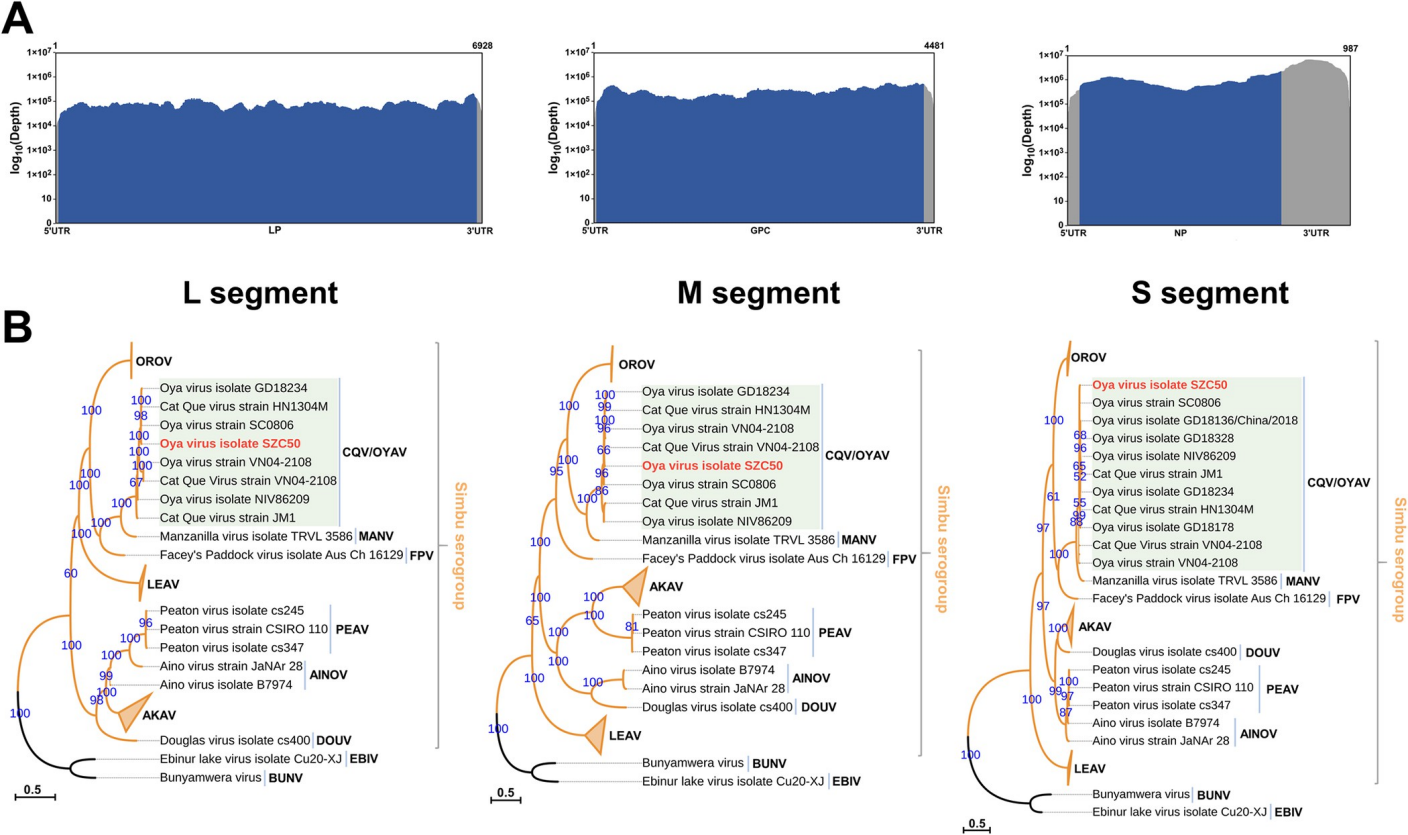
(A) The sequencing depth and the coverage of L, M and S segment of OYAV SZC50. The X-axis and Y-axis indicates position and sequencing depth, respectively. (B) Phylogenetic analyses of viruses in Simbu serogroup and *Bunyamwera orthobunyavirus*. The maximum likelihood phylogenetic tree was constructed based on ORFs from the S, M, and L segments of viruses. The ultrafast bootstrap was 5000 replicates. The best-fit model was estimated as the TVM+F+R3 for the S segment, GTR+F+I+G4 for the M segment, and GTR+F+I+G4 for the L segment, according to Bayesian information criterion (BIC). UTR: untranslated region; LP: L protein; GPC: glycoprotein precursor; NP: nucleoprotein.

Next-generation sequencing data of the OYAV SZC50 inoculated cell culture contained a total of 32,094,194 cleaned reads. After *de novo* assembly and alignment analysis, three of the contigs best hit to the S, M, and L segment of OYAV isolate SC0806, respectively. Each contig has larger than 10^5^ × sequencing depth and 100% coverage of predicted region (Fig 2A). The sequences were further confirmed by PCR and RACE. The complete genome of OYAV SZC50 was acquired and deposited in Genbank (S: OP672242, M: OP672243, L: OP672244), and the sequences were also available in S3 Table.

According to BLASTN results, the ORFs of the S, M, and L segments of SZC50 were >98% identical to those of OYAV SC0806 (Table 1). The ORF of the SZC50 S segment was 702 nucleotides (nt) in length and encoded a nucleocapsid protein (N) of 233 amino acids (aa). The protein had an isoelectric point [pI] of 9.59. The M segment ORF of SZC50 was 4,302 nt in length and encoded a polyprotein (1,433 aa and pI = 8.07) with a cleavable signal peptide. The ORF of L segment of SZC50 was 6,786 nt long. It encoded a large polypeptide (2261 aa in length) with a pI of 6.94.

**Table 1:** Features of ORFs encoded by OYAV SZC50.

Virus name	Isolation	Segment of genome	ORF length (nt|aa)	Protein mass (kDa)	Isoelectric point	Signal peptide	Top BLASTN match	E value	Identity	Accession
Oya virus	SZC50	S	702**|**233	26.41	9.59	-	Oya virus strain SC0806 segment S	0	98.68%	JX983192.1
		M	4302**|**1433	163.05	8.07	+	Oya virus strain SC0806 segment M	0	98.68%	JX983193.1
		L	6786**|**2261	264.23	6.94	-	Oya virus strain SC0806 segment L	0	98.8%	JX983194.1

To validate the relationship of SZC50 in this group, phylogenetic analyses of ORFs from the S, M, and L segments of viruses in Simbu serogroup were performed. SZC50 clustered with viruses belong to species *Orthobunyavirus catqueense* (CQV/OYAV) (Fig 2B). Multiple alignments of nucleotide and aa sequences were performed between SZC50 and the other CQV strains (S1 Fig). SZC50 shared the highest nucleotide and aa homology with OYAV SC0806. The findings confirmed that SZC50 belonged to *Orthobunyavirus catqueense* and represented a new isolate of OYAV.

### 3.3. Seroprevalence study for OYAV SZC50

**Fig 3:**
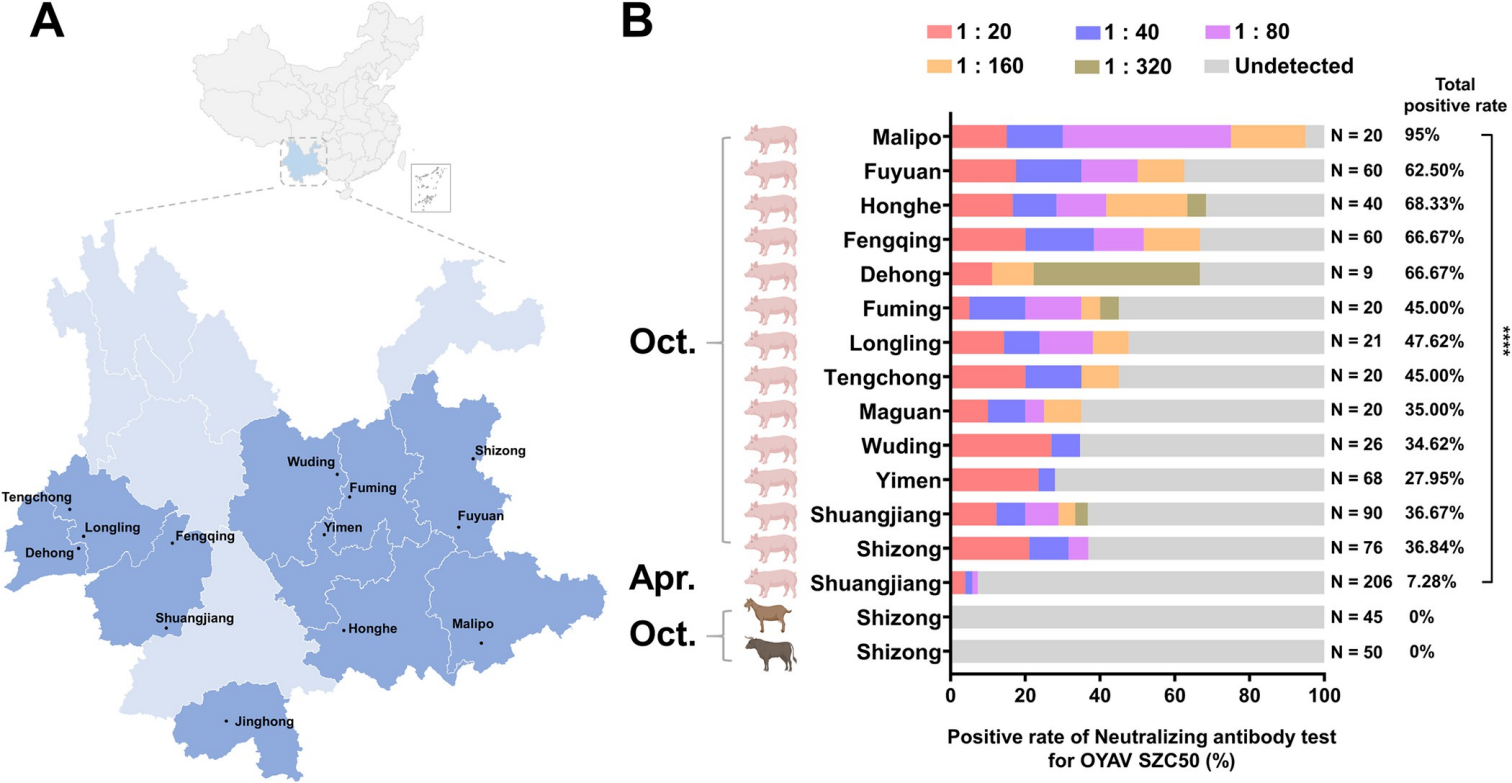
Serology of OYAV SZC50 in three domestic animals (pigs, cattle, and sheep) in different cities in Yunnan, China. (A) The sample sites in Yunnan. (B) Positive rates of OYAV SZC50 antibody in three domestic animal serum collected in April or October in 2019. ****: *P* < 0.00001. The source of the basemap shapefile were from https://www.webmap.cn/store.do?method=store&storeId=2.

In 2019, 831 serum samples (736 pigs, 45 cattle, and 50 sheep) were gathered from 13 cities in Yunnan (Fig 3). The OYAV SZC50 neutralizing antibody titer was ≥ 1: 20 in 270 serum samples, and the positive rate was 32.49% (270 pigs, 0 cattle, and 0 sheep) (Fig 3B). The positive rates of OYAV SZC50 antibody in the pig serum collected in October ranged from 27.95% to 95% in 13 sampling sites. However, the positive rate in the pig serum collected in April was just 7.28%.

To assess differences in OYAV SZC50 neutralizing antibody titers in porcine serum samples, the positive serum neutralizing antibody titers were divided into three groups: low titer (1:20 to 1:40), medium titer (1: 80 to 1:160), and high titer (> 1:320). Most of the neutralizing antibody titers in pig serum were 163 in the low titer group, accounting for 60.37% of the positive serum, 96 in the medium titer group, accounting for 35.56% of the positive serum, and 11 in the high titer group, accounting for 4.07% of the positive serum (Fig 3B).

### 3.4. Pathogenicity of OYAV SZC50 in mice

**Fig 4:**
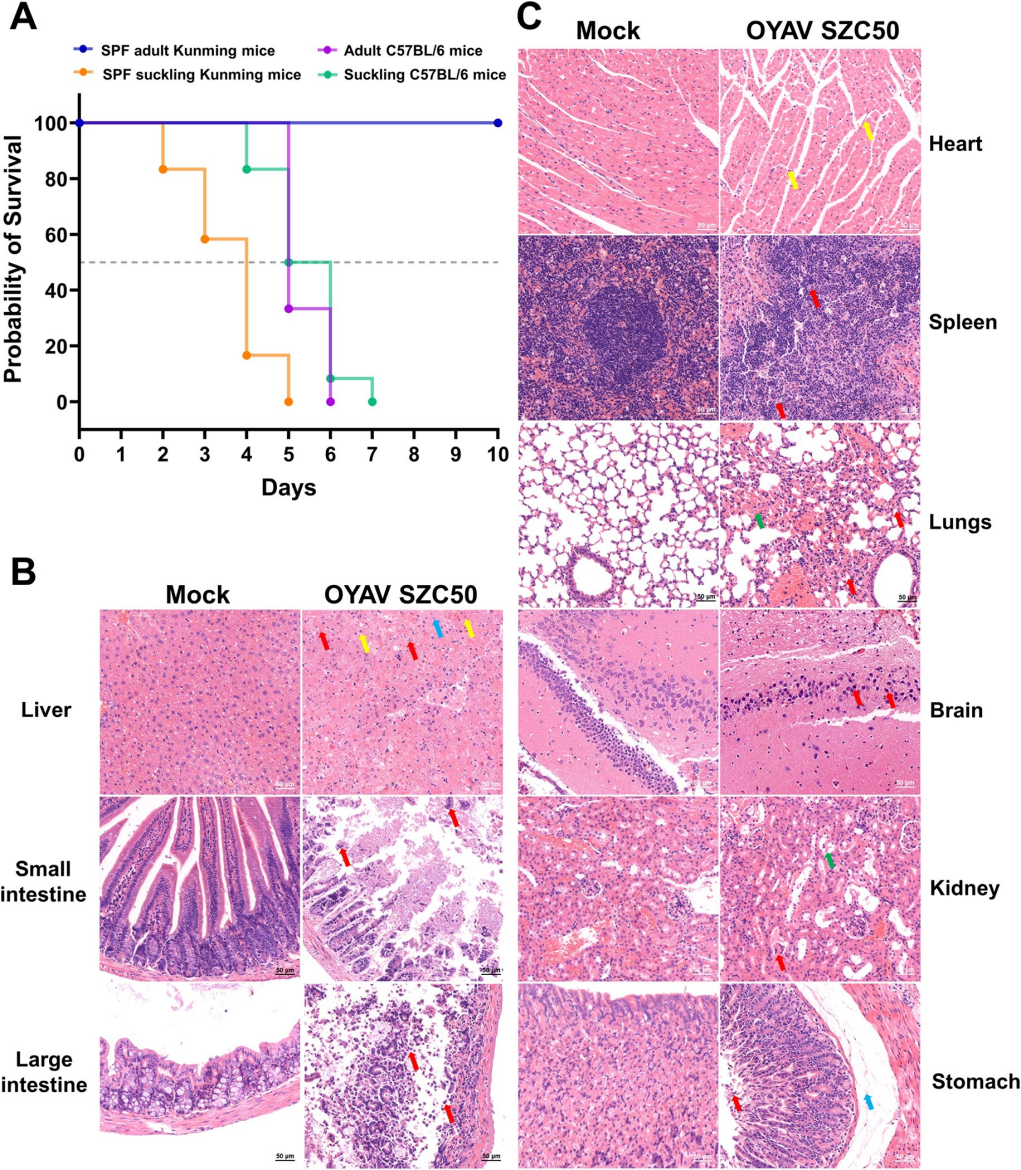
Survival rate and histopathological features of infected mice. (A) Survival rate of suckling and adult of Kunming and C57BL/6 mice infected with OYAV SZC50. (B and C) Histopathological features in the organs of C57BL/6 mouse infected by intraperitoneal inoculation. Liver: showed nuclear pyknosis and deep staining (red arrows); Small intestine: lamina propria was exposed, and numerous shedding tissues were visible in the intestinal lumen (red arrow). Large intestine: many epithelial cells had eroded and the lamina propria exposed (red arrow). Heart: individual inflammatory cells (yellow arrows) were found infiltrating the myocardial space. Spleen: the nuclei of cells were fragmented, pyknotic, and heavily stained (red arrow). Lungs: alveolar wall thickening was accompanied by alveolar atrophy (red arrow) and red blood cells filled a portion of the alveolus (green arrow). Brain: nuclei were condensed and stained (red arrows). Kidney: dilated cysts (red arrow) and some renal tubular epithelial cells had fallen off (green arrow). Stomach: many epithelial cells were eroded and shedding (red arrow), and mild edema was evident in the submucosal layer, enlarged tissue spaces, and fibrous tissue hyperplasia (green arrow).

After being inoculated with OYAV SZC50, adult Kunming mice survived normally, but all suckling Kunming mice, and adult and suckling C57BL/6 mice died by 5, 6, and 7 days post infection (dpi), respectively. Suckling Kunming mice became ill (milk refusal, segregation, convulsions, and other behaviors) on the second day and died. By the fifth day, 66.67% of the adult C57BL/6 mice had died, whereas 85% of the suckling mice had died by the fourth day (Fig 4A).

By the fifth day, one adult mouse infected with OYAV SZC50 and one control adult mouse were dissected to evaluate the pathological alterations of various tissues and organs. The liver color of the infected mouse was dark yellow. There was no food in the stomach, which was white in color. Pathological analysis of sections, the liver, small intestine, and large intestine of mice infected with OYAV SZC50 were severely damaged organs (Fig 4B). Heart, spleen, lungs, brain, kidney, and stomach displayed slight and moderate damage, respectively (Fig 4C).

Liver tissue structure of mice infected with OYAV SZC50 was severely aberrant. Degeneration and necrosis of liver cells were evident (nuclear pyknosis and deep staining are indicated by red arrows). In some liver cells, mild edema, cell swelling and light staining of the cytoplasm light staining were evident (indicated by blue arrows). A small amount of inflammatory cell infiltration was observed in the liver parenchyma (yellow arrows).

In the small intestine, many epithelial cells that had eroded and shed from the mucosal layer were observed. As well, lamina propria was exposed, and numerous shedding tissues were visible in the intestinal lumen (red arrow).

In the large intestine, the visible mucosal layer structure was incomplete, with a disordered arrangement. Many epithelial cells had eroded and the lamina propria exposed (red arrow).

In the heart, the myocardial tissue structure was slightly abnormal, with myocardial fibers arranged loosely. Individual inflammatory cells were found infiltrating the myocardial space (yellow arrows).

In the spleen, a few and erratically distributed red intramedullary lymphocytes were evident and there is no neutrophil infiltration. Some of the lymphocytes in the splenic nodules were necrotic, and the nuclei of cells were fragmented, pyknotic, and heavily stained (red arrow).

In the lungs, alveolar wall thickening was accompanied by alveolar atrophy (red arrows), blurring of the border of individual alveoli, and an abundance of proliferating alveolar epithelial cells, especially in the alveolar wall. Red blood cells filled a portion of the alveolus (green arrow).

In the brain, some neurons were degenerated, and nuclei were condensed and stained (red arrows).

In the kidney, individual glomerular atrophy, decreased number of mesangial cells in the glomerulus, dilated cysts, and a small amount of protein mucus visible in cysts were observed (red arrow). Some renal tubular epithelial cells had fallen off (green arrow).

Finally, in the stomach, epithelial cells in the mucosal layer displayed a disordered arrangement, and many epithelial cells were eroded and shedding (red arrow). Mild edema was evident in the submucosal layer, enlarged tissue spaces, and fibrous tissue hyperplasia (green arrow).

### 3.5. Pathogenicity of OYAV SZC50 to chicken embryos

**Fig 5:**
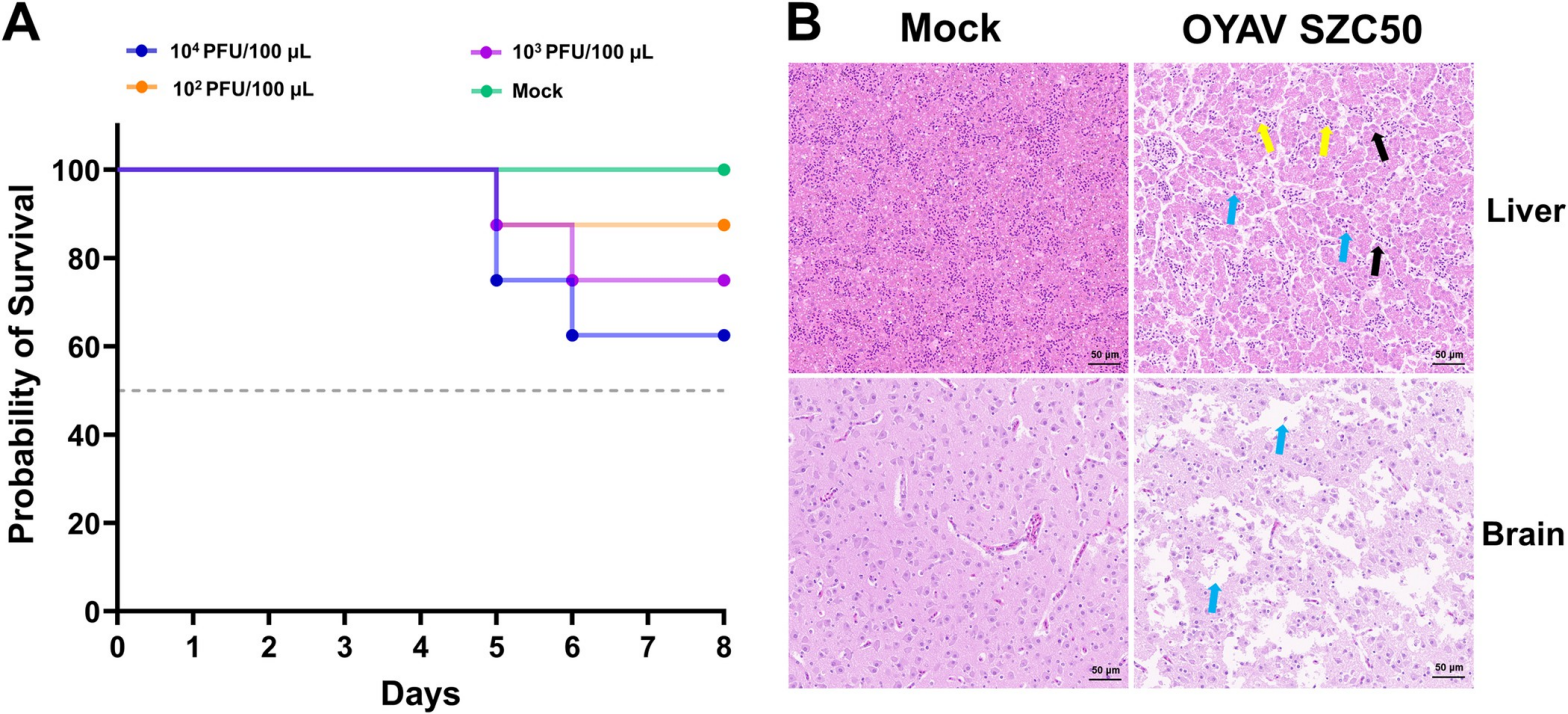
The survival rate and histopathological features of chicken embryo infected with OYAV SZC50. (A) The survival rate of chicken embryos (11-day-old) after inoculation of the allantoic cavity with 100 μL of the three viral titers (10^4^, 10^3^, and 100 PFU) of OYAV SZC50. (B) Histopathological features in the liver and brain of OYAV-infected chicken embryos by inoculation with OYAV SZC50 (100 μL containing 100 PFU) in the allantoic cavity. In liver tissue, edema of some cells, cell swelling, light staining of the was evident, as shown by black arrows. The yellow and blue arrow indicate hematopoietic stem cells and lymphocytes, respectively. In brain tissue, mild edema was evident in the brain parenchyma, the tissue gap increased, the cells displayed a disordered arrangement, and the structure was loose, as shown by blue arrows.

To determine the effect of virus titer of OYAV SZC50 on the survival rate of chicken embryos, the three different virus doses of OYAV SZC50 (10^4^, 10^3^, and 100 PFU) were injected into the allantoic cavity of 11-day-old chicken embryos. The demise of chicken embryos occurred primarily on the 5 and 6 days post-inoculation (Fig 5A). The death rate rose, albeit not dramatically, with increasing virus dose. The mortality rate in all experimental groups was < 50%. In the control group, no chick embryos died (Fig 5A). Anatomical examination of chicken embryos that died by day 5 revealed that each group experienced varying degrees of hemorrhage. The body size of chicken embryos infected with 10^4^ PFU OYAV SZC50 was much less than chicken embryos infected with the other virus doses and uninfected embryos. Bleeding was the most evident anatomical change, bodies were slightly transparent and covered in mucus, and brain tissue was pasted together (S5 Fig).

On the fourth day, virus treated and control embryos that has died were dissected to assess the pathological alterations, including changes of brain and liver tissue. Tissue edema, weak connective tissue structure, and neuronal gap widening was more common in the brain, blood vessel congestion and hepatic cell steatosis were more common in the liver, and small spherical vacuoles were detected in the cytoplasm (Fig 5B).

## 4. Discussion

We isolated and sequenced the genome of a new OYAV isolate (SZC50), from midge samples and confirmed the probable host tropism in vitro. In addition, the structure of the OYAV SZC50 genome, its phylogenetic location, viral morphology, seroprevalence, and pathogenicity in animal models were determined. Our findings were the first clear evidence of an OYAV virus isolate in midges in Yunnan Province, China (S2 Table). Notably, high levels of OYAV SZC50 antibody might be present in pig serum samples from Yunnan, and the virus caused severe mortality in suckling mice.

Phylogenetic analyses revealed that OYAV SZC50 was closely related with viruses from the *Orthobunyavirus catqueense* species. This viral species currently contains 11 virus isolates (S2 Table), including OYAV SC0806 identified in 2004 from mosquitos (*Culex tritaeniorhynchus*) in Sichuan Province, China [16]; OYAV VN04-2108 and CQV VN04-2108, both discovered from *Culex* mosquitos in Vietnam [30]; OYAV HN1304M isolated from naturally occurring *Culicoides* biting midges collected in 2013 in Hunan Province, China [31]; OYAV GD18234, OYAV GD18178, OYAV GD18328, and OYAV GD18136/China/2018 discovered from *Chironomus thummi* in Guangdong Province, China [17]; OYAV NIV86209 and CQV JM1, detected in humans or myna in India [13]; and an OYAV isolated in Vero cell cultures from the lungs of a pig in peninsular Malaysia [14]. The three OYAV isolates discovered from biting midges in Guangdong only possessed the S segment sequence and the viruses could not be isolated. The S, M, and L ORFs of OYAV SZC50 displayed extensive similarity with those of OYAV SC0806 (Table 1 and S1 Fig). Multiple amino acid sequence alignments between OYAV SZC50 and OYAV SC0806 revealed eight alterations in the M protein (aa349: D-E, aa571: R-K, aa638: M-T, aa901: S-L, aa1139: T-A, aa1401: F-V, and aa1421: R-K), compared to one change in the N protein (aa120: K-E) and four alterations in the L protein (aa487: E-G, aa1202: A-V, aa1782: R-K, aa2055: V-A) (S2, S3, and S4 Figs). The Gc and Gn glycoproteins form capsomeric projections or spikes on the virion surface and had a critical role in viral entry, assembly, and morphogenesis. Both glycoproteins are encoded by the M segment ORF [32]. Following infection or vaccination, the glycoproteins are both the principal target of host neutralizing antibody responses [33]. Whether these amino acid substitutions affect viral replication and virulence needs further study.

Based on the findings of viral proliferation curves in several cell lines, it was determined that PK15 pig kidney derived cells are the best cell host for OYAV SZC50 amplification (Fig 1B). Notably, OYAV SZC50 could also proliferate effectively in human (HeLa) and monkey (MA104) cell lines. Furthermore, serological data from pigs indicated that a significant proportion of OYAV SZC50 antibody (> 30%) was detected in Yunnan pig populations, with the positive rate of OYAV SZC50 antibody in pigs from reaching 95% (Fig 3). In contrast, this antibody was not detected in cattle or sheep serum. Since there is a probability of other orthobunyaviruses co-circulation in Yunnan, this data does not rule out the possibility of a cross-reaction with other related viruses in the PRNT test, which is not excluded the possibility for cross-reactivity with other related viruses. In our future work, it needs to carry out virus isolation in serum samples from sick pigs in the acute phase, the preparation of specific monoclonal antibodies for the OYAV, and nucleic acid detection of sick pigs in the acute phase in order to further confirm the positive rate of OYAV in the pig population in this area. A previous study also revealed a high titer of immunoglobulin M (IgM) and IgG antibodies of OYAV SC0806 in pigs reared in Sichuan Province, China [16]. Pigs may be the principal mammalian host of CQV [30,34]. Moreover, anti-CQV IgG antibody was described in two of 883 human serum samples examined in India, whereas swine serum samples were reportedly negative for anti-CQV IgG antibodies [35]. The collective findings indicate that it is possible that OYAV has been prevalent in the pig population in several areas of China, notably in Yunnan. The risks of a CQV epidemic are comparable to those of other vector-borne viruses [36] and the potential risk of OYAV infection in humans cannot be ignored.

The pathogenicity of CQVs in animal models is unknown. Previous studies examined the Oropouche virus, an orthobunyavirus in the Simbu serogroup, in suckling mice and hamsters following intracerebral inoculation [37,38]. Chicken embryo models have been used to investigate the pathogenicity and teratogenicity of Simbu viruses, including Schmallenberg virus (SBV) and Akabane virus (AKAV), which also belong to the Simbu serogroup [39]. We selected SPF Kunming mice, C57BL/6 mice lacking the interferon α/β receptor, and chicken embryos to establish the pathogenicity of OYAV SZC50. All control groups had no clinical symptoms and pathological changes. Like OROV, suckling mice were also susceptible to OYAV SZC50 infection established by intracerebral inoculation. The lethality of OYAV SZC50 to 1-day-old SPF Kunming suckling mice was fast. OYAV SZC50 produced lethality rates of 100% for SPF Kunming suckling mice and suckling C57BL/6 mice (Fig 4A). Notably, although adult C57BL/6 mice were similarly highly vulnerable to OYAV SZC50 infection introduced via intraperitoneal injection, SPF adult Kunming mice survived. The viral load in the blood of SPF adult Kunming mice was consistently maintained at a low level (S4 Table). Moreover, the neutralizing antibody titers in these mice were typically 1:40 and 1:80, consistent with the findings that the neutralizing antibody titers of the pigs were usually low (S5 Table and Fig 3B). Compared with Ebinur Lake virus belonging to *Orthobunyavirus*, which caused substantial mortality in immunocompetent, 6-8-week-old BALB/c mice, OYAV SZC50 may be less virulent (Fig 4A) [40]. After inoculation with OYAV SZC50 in the allantoic cavity, some chicken embryos died. The highest lethality rate was 37.5% (Fig 5A), which is slightly higher than that observed in SBV or AKAV-infected chicken embryos [39]. In addition, chicken embryos infected with AKAV and SBV show obvious stunted growth and musculoskeletal malformations. In the future, it will be necessary to investigate whether OYAV SZC50 has a similar effect on chicken embryos.

Pathological evaluations revealed that the heart tissues of infected adult mice did not display pathological changes, while other diseased organs (liver, spleen, lungs, small intestine, large intestine, brain, kidney, and stomach) displayed varying degrees of pathological changes (Fig 4B and 4C). In the brains of adult C57BL/6 mice infected with OYAV SZC50, gaps formed around many neuronal cells, and local hippocampus pyramidal cells were distributed unevenly. Tissue edema, weak connective tissue structure, and neuronal gap widening were also observed in the brains of OYAV SZC50-infected chicken embryos (Fig 5B). Previous histological findings revealed lesions (meningo-encephalomyelitis, perivascular cuffing of lymphocytes and macrophages, neuronal degeneration, and gliosis) in the central nervous system of IFNAR-/- mice infected with Simbu viruses [41]. Similar findings were described after intracerebral injection of mice with AKAV, another member of the serogroup [42]. Further histopathology and immunohistochemical staining results will be required to detail the effects of OYAV SZC50 on the nervous system of the animal models.

## Supporting information

S1 Fig Comparison of nuclear acid and amino acid consensus sequences nucleotide and amino acid sequences of SZC50 with the other CQVs. (TIF)

S2 Fig Multiple alignments of amino acid sequences of SZC50 N (A) and NSs (B) protein with those of other CQVs. (PDF)

S3 Fig Multiple alignments of amino acid sequences of SZC50 M protein with the other CQVs. (PDF)

S4 Fig Multiple alignments of amino acid sequences of SZC50 L protein with the other CQVs. (PDF)

S5 Fig Gross morphology of chicken embryos after inoculation with 100 μL of each of the three viral titers of OYAV SZC50 (104, 103, and 100 PFU) in the allantoic cavity. (TIF)

S1 Table Virus-specific and RACE primers used to confirm viral genome. (DOCX)

S2 Table Virus species used in the present study. (XLSX)

S3 Table The sequences of L, M and S segment of OYAV SZC50. (XLSX)

S4 Table OYAV SZC50 CT values detected in the blood of SPF adult Kunming mice inoculated intraperitoneally with 500 μL SZC50 solution (100 PFU/100 μL) on different days. (DOCX)

S5 Table OYAV SZC50 neutralizing antibody titers detected in the blood of SPF adult Kunming mice inoculated intraperitoneally with 500 μL SZC50 solution (100 PFU/100 μL) on different days. (DOCX)
