# Supplementary figures and images for: In vivo and in vitro characterization of a new Oya virus isolate from Culicoides spp. and its seroprevalence in domestic animals in Yunnan, China
Source: PLoS Negl Trop Dis. 2023 Jun 15;17(6):e0011374. doi: 10.1371/journal.pntd.0011374 (PMC10306208; doi:10.1371/journal.pntd.0011374)

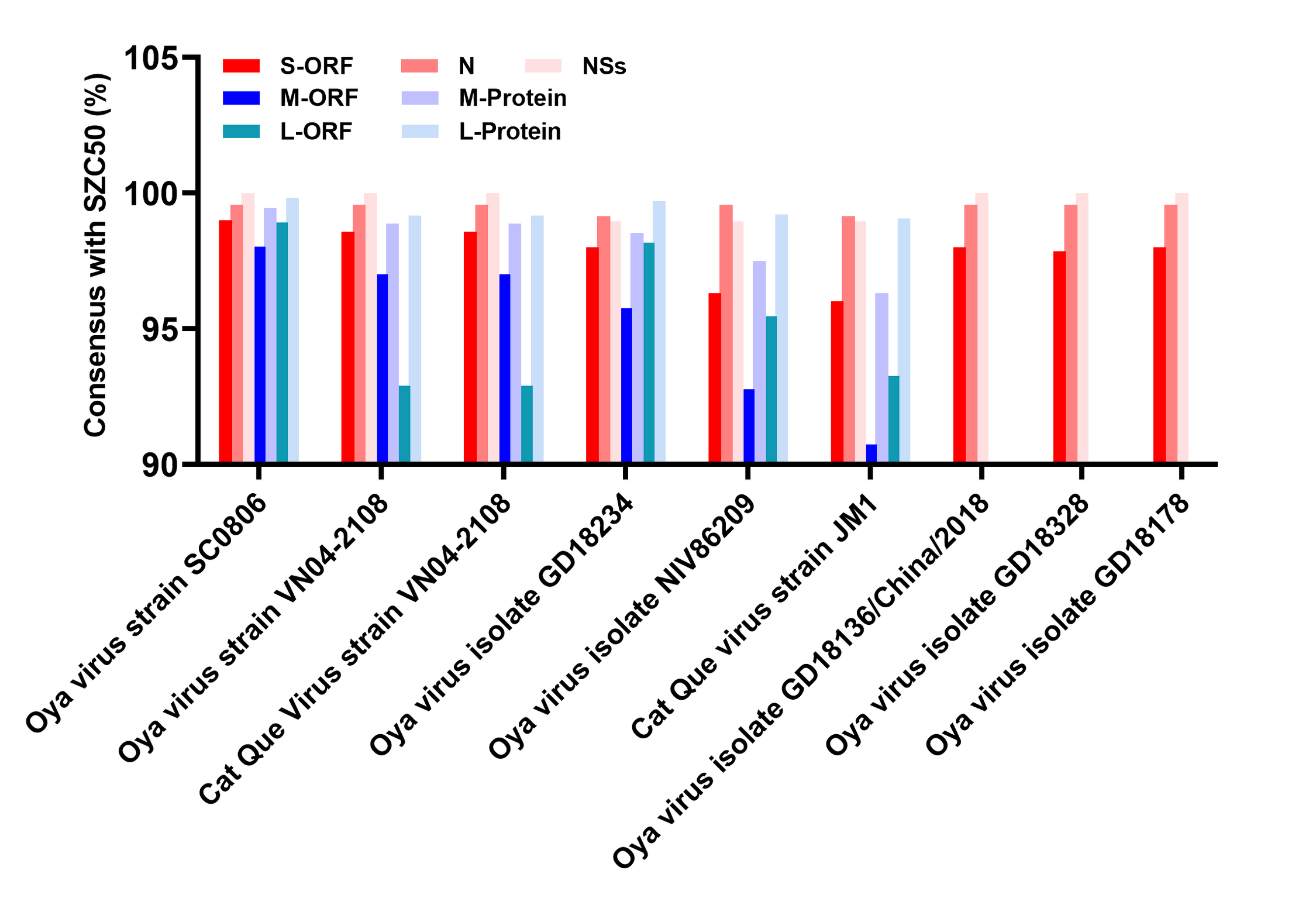

Supplement: S1 Fig — (TIF) [file pntd.0011374.s001.tif]

**A**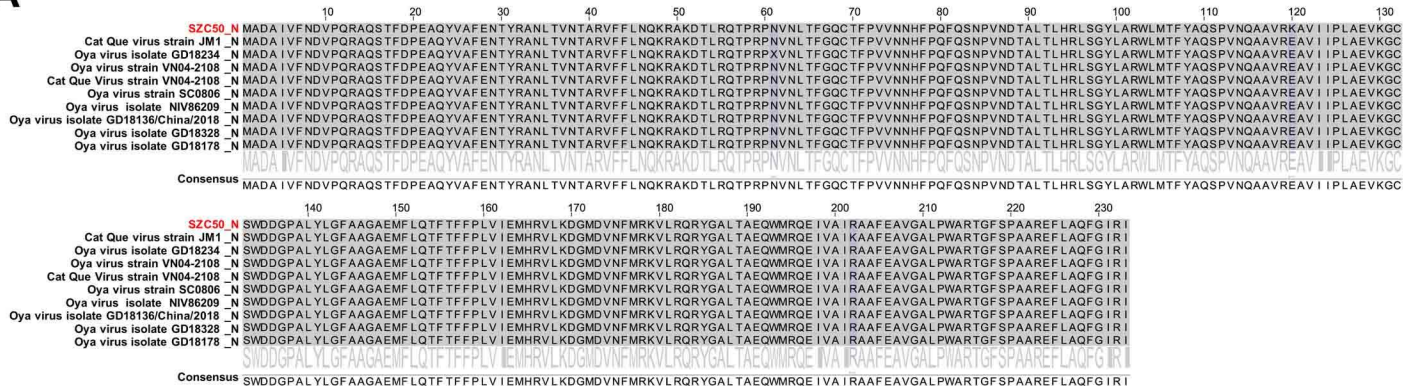**B**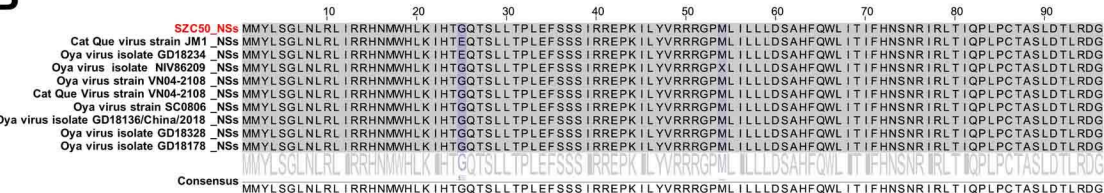

Supplement: S2 Fig — (PDF) [file pntd.0011374.s002.pdf]

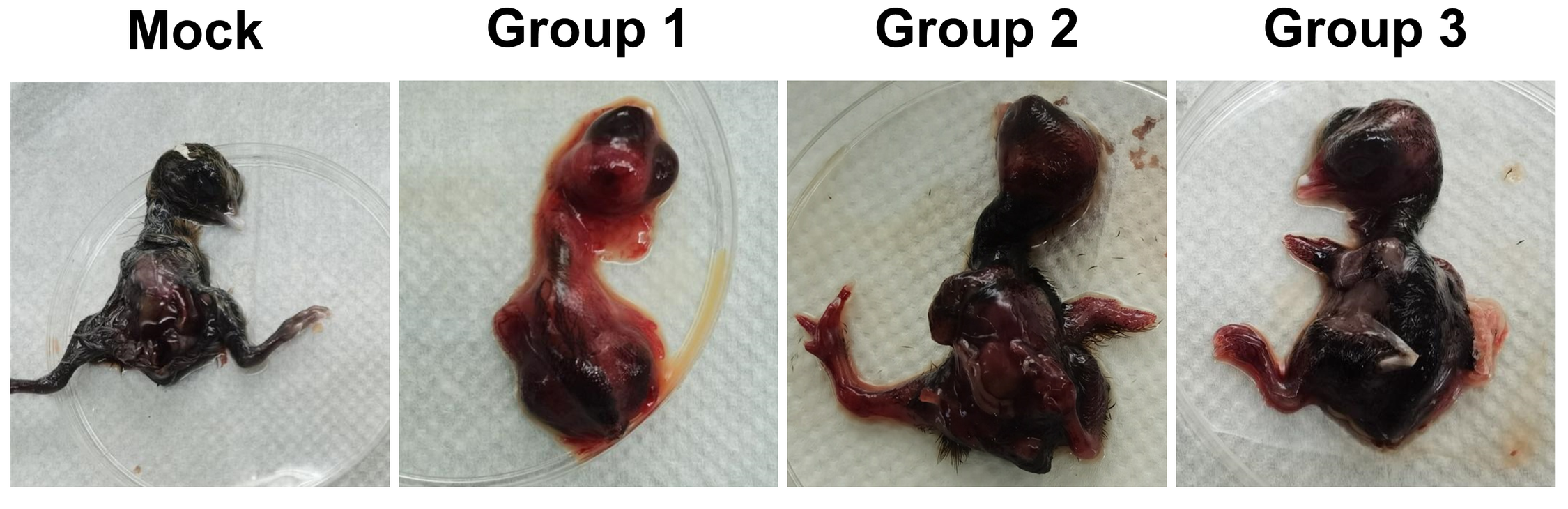

Supplement: S5 Fig — (TIF) [file pntd.0011374.s005.tif]
