# Supplementary material for: In vivo and in vitro characterization of a new Oya virus isolate from Culicoides spp. and its seroprevalence in domestic animals in Yunnan, China
Source: PLoS Negl Trop Dis. 2023 Jun 15;17(6):e0011374. doi: 10.1371/journal.pntd.0011374 (PMC10306208; doi:10.1371/journal.pntd.0011374)
Supplement: S3 Fig — (PDF) [file pntd.0011374.s003.pdf]

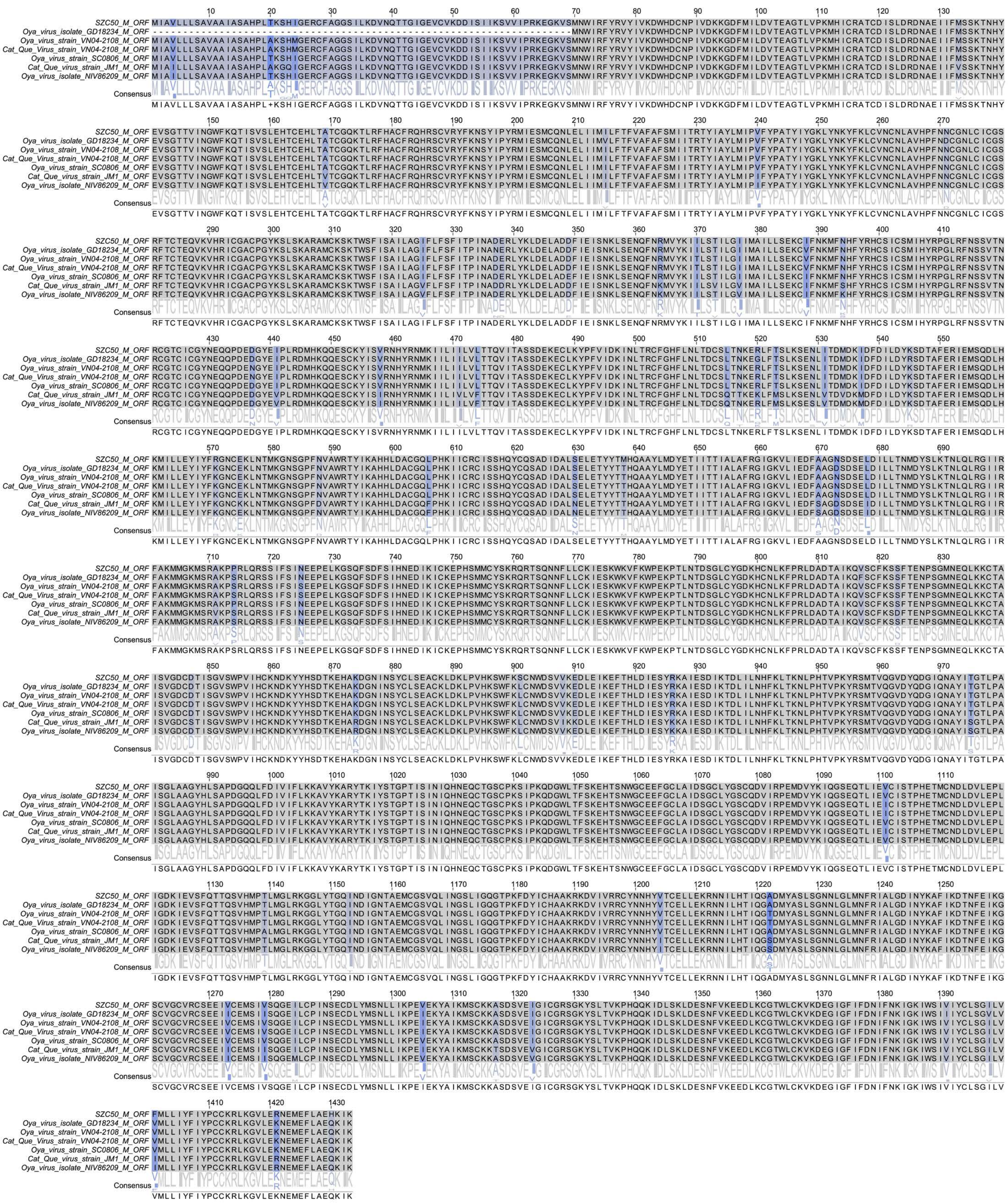
SZC50_M_ORF
Oya_virus_isolate_GD18234_M_ORF
Oya_virus_strain_VN04-2108_M_ORF
Cat_Que_Virus_strain_VN04-2108_M_ORF
Oya_virus_strain_SC0806_M_ORF
Cat_Que_virus_strain_JM1_M_ORF
Oya_virus_isolate_NIV86209_M_ORF
Consensus
MIAVLLLSAVAAIASAHPL+KSHIGERCFAGGSILKDVNQTTGIGEVCVKDDISIIKSVVIPRKEGKVSMNWIRFYRVYIVKDWHDCNPIVDKKGDFMILDVTEAGTLVPKMHICRATCDISLDRDNAEIIFMSSKTNHY
EVSGTTVINGWFKQTISVSLEHTCEHLTATCGQKTLRFHACFRQHRSCVRYFKNSYIPYRMIESMCQNLELIIMILFTFVAFAFSMIITRTYIAYLMIPVFYPATYIYGKLYNKYFKLCVNCNLAVHPFNNCGNLCICGS
RFTCTEQVKVHRICGACPGYKSLSKARAMCKSKTWSFISAILAGIFLFSFITPINADERLYKLDELADDFIEISNKLSENQFNRMVYKIILSTILGIIMAILLSEKCIFNKMFNHFYRHCSICSMIHYRPGLRFNSSVTN
RCGTCICGYNEQQPDEDGYEIPLRDMHKQQESCKYISVRNHYRNMKIILILVLTTQVITASSDEKECLKYPFVIDKINLTRCFGHFLNLTDCSLTNKERLFTSLKSENLITDMDKIDFDILDYKSDTAFERIEMSQDLH
KMILLEYIYFKGNCEKLNTMKGNSGPFNVAWRTYIKAHHLDACGQLPHKIICRCISSHQYCQSADIDALSELETYYTTHQAAYLMDYETIITTIALAFRGIGKVLIEDFAAGNSDSELDILLTNMDYSLKTNLQLRGIIR
FAKMMGKMSRAKPSRLQRSSIFSINEEPELKGSQFSDFSIHNEDIKICKEPHSMMCYSKRQRTSQNNFLLCKIESKWKVFKWPEKPTLNTDSGLCYGDKHCNLKFPRLDADTAIKQVSCFKSSFTENPSGMNEQLKKCTA
ISVGDCDTISGVSWPVIHCKNDKYYHSDTKEHAKDGNINSYCLSEACKLDKLPVHKSWFKLCNWDSVVKEDLEIKEFTHLDIESYRKAIESDIKTDLILNHFKLTKNLPHTVPKYRSMTVQGVDYQDGIQNAYITGTLPA
ISGLAAGYHLSAPDGQQLFDIVIFLKKAVYKARYTKIYSTGPTISINIQHNEQCTGSCPKSIPKQDGWLTFSKEHTSNWGCEEFGCLAIDSGCLYGSCQDVIRPEMDVYKIQGSEQTLIEVCISTPHETMCNDLDVLEPL
IGDKIEVSFQTTQSVHMPTLMGLRKGGLYTGQINDIGNTAEMCGSVQLINGSLIGQGTPKFDYICHAAKRKDVIVRRCYNNHYVTCELLEKRNNILHTIQGADMYASLSGNNLGLMNFRIALGDINYKAFIKDTNFEIKG
SCVGCVRCSEEIVCEMSIVSQGEILCPINSECDLYMSNLLIKPEIEKYAIKMSCKKASDSVEIGICGRSGKYSLTVKPHQQKIDLSKLDESNFVKEEDLKCGTWLCKVKDEGIGFIFDNIFNKIGKIWSIVIYCLSGILV
VMLLIYFIYPCCKRLKGVLEKNEMEFLAEQKIK
