# Supplementary material for: In vivo and in vitro characterization of a new Oya virus isolate from Culicoides spp. and its seroprevalence in domestic animals in Yunnan, China
Source: PLoS Negl Trop Dis. 2023 Jun 15;17(6):e0011374. doi: 10.1371/journal.pntd.0011374 (PMC10306208; doi:10.1371/journal.pntd.0011374)
Supplement: S1 Table — (DOCX) [file pntd.0011374.s006.docx]

**S2 Table. Virus-specific and RACE primers used to confirm viral genome**

| **Primer** | **Genome position** | **Oligonucleotide (5'→3')** | **Orientation** | **Application** |
| --- | --- | --- | --- | --- |
| OYAV-L-1F | 1-48 | AGTAGTGTACCCCTAGGTTACAACATACAACGATTCTAAGAACATATC | → | To confirm viral genome |
| OYAV-L-1R | 2224-2249 | GCCGGGAAACCAGATTGACCTTAGAT | ← |  |
| OYAV-L-2F | 2229-2254 | AGGTCAATCTGGTTTCCCGGCAAAGT | → |  |
| OYAV-L-2R | 4854-4880 | ACTCCTCATTTCTGGCATCCTGCAAGC | ← |  |
| OYAV-L-3F | 4858-4887 | GCAGGATGCCAGAAATGAGGAGTTTAAAAT | → |  |
| OYAV-L-3R | 6895-6928 | AGTAGTGTGCCCCTAGGAACATTAATATACTTAC | ← |  |
| OYAV-M-1F | 1-39 | AGTAGTGTACTACCACATACAACAAACTTTTCAGAGAAT | → |  |
| OYAV-M-1R | 2152-2181 | GGCTTAGCTCTAGACATTTTCCCCATCATC | ← |  |
| OYAV-M-2F | 2161-2192 | GAAAATGTCTAGAGCTAAGCCACCAAGACTGC | → |  |
| OYAV-M-2R | 4437-4481 | AGTAGTGTGCTACCACGTACAAACATTCAAATTTATTTAATTAGC | ← |  |
| OYAV-S-1F | 1-32 | AGTAGTGTACTCCACAATTCAAAAACTTAAAG | → |  |
| OYAV-S-1R | 960-985 | AGTAGTGTGCTCCCAATTCAAAGATG | ← |  |
| OYAV-L-5R | 345-372 | CTCGGGCCTCCCGTGCACTTCTTTCATC | ← | 5' RACE |
| OYAV-M-5R | 531-558 | CCGCATGTGGCAGTTAAATGCTCGCAAG | ← |  |
| OYAV-S-5R | 369-395 | AGCTGCTTGGTTGACTGGGCTCTGGGC | ← |  |
| OYAV-L-3F | 6338-6365 | CAATACCAAGGGCGAGAGGGGAATGACC | → | 3' RACE |
| OYAV-M-3F | 4020-4047 | GTGGGCGGTCAGGAAAATACAGCTTAAC | → |  |
| OYAV-S-3F | 659-685 | TGAGGCTGTTGGCGCACTCCCTTGGGC | → |  |
