# Supplementary material for: In vivo and in vitro characterization of a new Oya virus isolate from Culicoides spp. and its seroprevalence in domestic animals in Yunnan, China
Source: PLoS Negl Trop Dis. 2023 Jun 15;17(6):e0011374. doi: 10.1371/journal.pntd.0011374 (PMC10306208; doi:10.1371/journal.pntd.0011374)
Supplement: S4 Table — (DOCX) [file pntd.0011374.s009.docx]

S4 Table. OYAV SZC50 CT values detected in the blood of SPF adult Kunming mice inoculated intraperitoneally with 500 μL SZC50 solution (100 PFU/100 μL) on different days. Every 3 days, two tubes (100 μL) of whole blood were obtained from each adult mouse. Blood was collected continuously for 41 days following the inoculation. One tube was used to extract RNA by using TRIzol™ Reagent, and the other tube was used to separate serum. RNA was utilized to determine the duration and titer of viremia.

| Laboratory animal Number | Day 0 | Day 3 | Day 6 | Day 9 | Day 13 | Day 16 |
| --- | --- | --- | --- | --- | --- | --- |
| 1 | - | 32.98 | 34.25 | - | - | - |
| 2 | - | 30.43 | 32.51 | 38.42 | - | - |
| 3 | - | 32.56 | 36.32 | - | - | - |
| 4 | - | 31.91 | 35.18 | - | - | - |
| 5 | - | 33.7 | - | - | - | - |
| 6 | - | 31.53 | 35.85 | - | - | - |
