# Supplementary material for: In vivo and in vitro characterization of a new Oya virus isolate from Culicoides spp. and its seroprevalence in domestic animals in Yunnan, China
Source: PLoS Negl Trop Dis. 2023 Jun 15;17(6):e0011374. doi: 10.1371/journal.pntd.0011374 (PMC10306208; doi:10.1371/journal.pntd.0011374)
Supplement: S5 Table — (DOCX) [file pntd.0011374.s010.docx]

S5 Table. OYAV SZC50 neutralizing antibody titers detected in the blood of SPF adult Kunming mice inoculated intraperitoneally with 500 μL SZC50 solution (100 PFU/100 μL) on different days. Serum antibody was detected by Indirect Immunofluorescence assay (IFA).

| Laboratory animal Number | Day 0 | Day 3 | Day 6 | Day 9 | Day 13 | Day 16 | Day 20 | Day 23 | Day 27 | Day 34 | Day 41 |
| --- | --- | --- | --- | --- | --- | --- | --- | --- | --- | --- | --- |
| 1 | - | - | - | 1:40 | 1:80 | 1:40 | 1:80 | 1:80 | 1:40 | 1:40 | 1:40 |
| 2 | - | - | - | 1:80 | 1:160 | 1:80 | 1:80 | 1:80 | 1:80 | 1:80 | 1:80 |
| 3 | - | - | 1:20 | 1:40 | 1:40 | 1:40 | 1:80 | 1:40 | 1:40 | 1:40 | 1:40 |
| 4 | - | - | 1:20 | 1:80 | 1:80 | 1:40 | 1:40 | 1:40 | 1:40 | 1:40 | 1:40 |
| 5 | - | - | 1:20 | 1:40 | 1:40 | 1:80 | 1:80 | 1:80 | 1:80 | 1:80 | 1:80 |
| 6 | - | - | 1:20 | 1:40 | 1:80 | 1:80 | 1:80 | 1:80 | 1:80 | 1:80 | 1:80 |
